# Methionine increases yolk production to offset the negative effect of caloric restriction on reproduction without affecting longevity in *C. elegans*

**DOI:** 10.18632/aging.102770

**Published:** 2020-02-06

**Authors:** Gaojian Zhou, Chuan Huang, Liu Xing, Le Li, Yan Jiang, Yuehua Wei

**Affiliations:** 1 Basic Medicine College, Central South University, Changsha, Hunan, People's Republic of China; 2 Hunan Gaocell Medical Technology Co. Ltd., Changsha, Hunan, People's Republic of China; 3 Reproductive and Genetic Hospital of CITIC-Xiangya, Changsha, Hunan, People's Republic of China; 4 ProMab Biotechnologies, Richmond, CA 94806, USA; 5 Shanghai Ninth People's Hospital, Shanghai Jiao Tong University, School of Medicine, Shanghai, People's Republic of China

**Keywords:** methionine, reproduction, calorie restriction (CR), side effect, vitellogenin

## Abstract

Caloric restriction (CR) or Dietary restriction (DR) is known to improve health and in many cases increases lifespan. However, its negative effect on reproduction has not been fully studied. Practicing CR/DR without adequate knowledge on its side effect may risk complications such as infertility, birth defect, or malnutrition. In this study, by using several CR strategies in *C. elegans*, we examine key functions of reproduction including embryonic development and larvae growth. We find that CR significantly decreases the survival of embryos and slows the growth of the offspring. We further determine that defect in oocyte but not sperm is responsible for the compromised reproduction under CR. Interestingly, adding methionine to the medium reverses the reproduction defects, but does not affect the long lifespan resulted from CR. The beneficial effect of methionine on reproduction requires the yolk protein vitellogenin. CR down-regulates vitellogenin expression, which can be reversed by supplementing methionine in the food. Lacking the yolk protein transport due to *rme-2* mutation blocks methionine’s beneficial effects. Our study has revealed a novel, methionine-mediated genetic pathway linking nutrient sensing to reproduction and suggested methionine as a potential food supplement to mitigate the side effect of CR.

## INTRODUCTION

Nutrition status during pregnancy can modulate gene transcription of embryos, therefore causing physiological and structural change in the offspring [1]. Dysregulation of maternal nutrition intake during pregnancy might predispose the individual to metabolic, endocrine, and cardiovascular diseases in postnatal life [2]. However, little information is available regarding how the information are transmitted from parents to affect physiological and pathological changes in the next generation.

Caloric restriction (CR) or dietary restriction (DR) is a method to decrease food intake without causing malnutrition. Increased lifespan has been observed in many types of calorie-restricted animals, including *C. elegans*, *Drosophila*, mice and rhesus monkeys, suggesting a common and highly conserved mechanism [3]. Importantly, restricting calorie uptake not only prolongs lifespan, but also significantly improves various health parameters in higher animals [4–7]. In human, CR can significantly improve health of patients with diabetes, cardiovascular disease and some cancers [8–10]. CR can also protect against neurodegenerative disease and improve memory in elderly humans [11, 12]. However, negative effect has also been observed. For example, CR can cause muscle loss [13], bone loss [14] and compromised immune response [15]. In large population studies of the Dutch famine during World War II, long-term undernutrition resulted in reduced birth weight, head circumference and mental and physical health in early adult life [16–18].

Methionine restriction (MetR), similar to CR, extends lifespan in yeast [19–21], flies [22, 23], worms [24] and rodents [25–27]. The mechanism remains not fully defined. However, studies have suggested that MetR could achieve the beneficial effect through the growth hormone/insulin-like growth factor 1 (GH/IGF1) pathway [25], the mitochondrial respiration pathway [28] and/or the transsulfuration pathway [29], among many others [30]. CR/DR effect in *Drosophila* has been attributed to some essential amino acid including methionine [31]. Methionine is especially important as it can specifically improve egg production in chicken [32] and milk production of dairy cows [33], suggesting a specific role in nutrient metabolism and reproduction.

Considering the direct effect of maternal nutrition on reproduction health, it is surprising that little studies have been shown to address the effect of CR on reproduction. Although lacking evidence of lifespan extension in human, CR/DR has become very popular and more and more people, especially young generations are practicing CR/DR. Therefore, accurate evaluation of CR/DR’s risk is needed to avoid potential side effects that might negatively impact reproductive health of relevant individuals.

In this study, we used *C. elegans* as the animal model to study the effect of CR/DR on reproduction system such as egg, sperm and embryonic development. We find that CR shows deleterious effect on reproduction including increased mortality rate of embryo and delayed growth of offspring. We further dissect the underlying mechanisms and find that the negative effect on reproduction is attributed to the yolk protein vitellogenin in the eggs. Male reproduction does not appear to be impaired by CR/ DR. The essential amino acid methionine can mitigate the negative effect of CR but leaving the extended lifespan unchanged. Our study provides important knowledge to better understand the side effect of CR on reproduction.

## RESULTS

### Methionine mitigates the reproduction defect caused by CR in *C. elegans*

**Figure 1:**
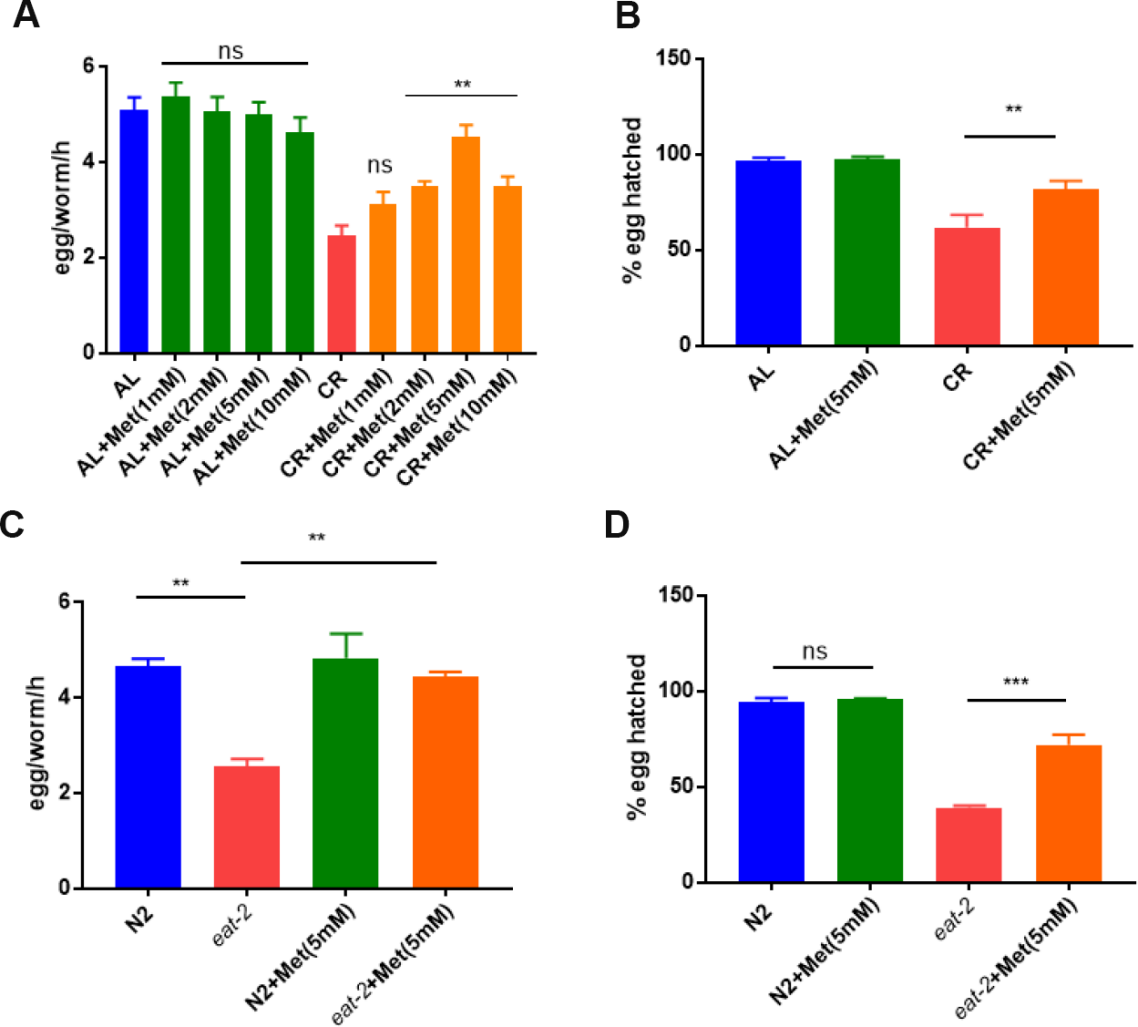
**Methionine supplementation prevents calorie restriction (CR) to reduce egg production and survival.** (**A**) Egg production was reduced by CR and rescued by methionine. Ad libido (AL) and CR conditions were achieved by plating 1X10^11^/mL and 1X10^8^/mL OP-50 bacteria, respectively, on solid nematode growth (NG) medium containing carbenicillin and kanamycin. Worms were raised on NG medium supplemented with indicated concentrations of methionine (Met) from hatching to day-1 adulthood. Worms (n>25) were allowed to lay eggs for 3 hours and egg production were evaluated in per worm per hour (egg/worm/h). Data were collected from 3 independent experiments. P values were obtained by t-test: ns, not significant; **, P<0.001. (**B**) Embryos survival rate was reduced b CR and rescued by methionine. Worms were raised on AL and CR conditions to day-1 adulthood and eggs were collected within 3 hours to obtained synchronized embryos. Hatching rate was determined by examining the number of dead eggs and larvae at L2/L3 stage. Data were collected from 3 independent experiments with each experiment examining n>100 animals. P values were obtained by t-test: **, P<0.001. (**C**) Egg production was reduced in CR model (*eat-2* mutant) and rescued by methionine. N2 wild-type and *eat-2(ad1116)* worms were raised on NG medium supplemented with and without 5mM methionine (Met) today-1 adulthood. Egg production was evaluated as in A. Data were collected from 3 independent experiments. P values were obtained by t-test: **, P<0.001. (**D**) Embryos survival rate was reduced in CR model (*eat-2* mutant) and rescued by methionine. N2 wild-type and *eat-2(ad1116)* worms were raised on NG medium supplemented with and without 5mM methionine (Met) today-1 adulthood. Embryo survival rate was determined as in C. Data were collected from 3 independent experiments. P values were obtained by t-test: ns, not significant; **, P<0.001.

Considering the important and specific roles of methionine in nutrient metabolisms, we were interested to know if methionine could mitigate the reproduction defect caused by calorie restriction. Calorie restriction is known to reduce offspring numbers in many organisms [34], a side effect that could be conserved in mammals. To understand the roles of methionine in growth and reproduction under CR/DR condition, we used CR/DR strategies established early in C. elegans [35]. To calorie restrict the animals, we diluted bacteria and cultured worms from L1 larvae stage on nematode growth (NG) medium plates supplemented with various concentrations of methionine (0, 1, 2, 5 and 10 mM). We found that CR/DR robustly reduced total egg production, which however was rescued by supplementing methionine at 2, 5, and 10 mM, with 5 mM achieving the best rescue (Figure 1A). To test if the rescue effect was specific to methionine, we also added cysteine, a sulfur-containing amino acid similar to methionine, and another 2 essential amino acids, threonine and leucine. However, at concentrations ranging from 1 to 10 mM, no rescue effect was observed for cysteine, threonine and leucine (Supplementary Figure 1). We also tested the cysteine precursor n-acetyl-cysteines (NAC), which has high water solubility. As shown in Supplemental Information Supplementary Figure 2, NAC slightly improved the egg production in both control and CR/DR groups in a dose-dependent manner, suggesting that NAC has no specific function in the CR/DR pathway. Second, we examined the effect of methionine on egg hatching. CR robustly decreased the egg hatching rate. Interestingly, such detrimental effect of CR was significantly mitigated by 5 mM methionine (Figure 1B). To confirm the observations, we also conducted similar experiments by using eat-2(ad1116) mutant. The eat-2 mutant has a reduced pharyngeal pumping rate, hence reduced food uptake, which makes it a widely-used CR/DR model [36]. Consistently, defect in egg production and hatching in eat-2 mutant were significantly improved by methionine (Figure 1C and 1D).

**Figure 2:**
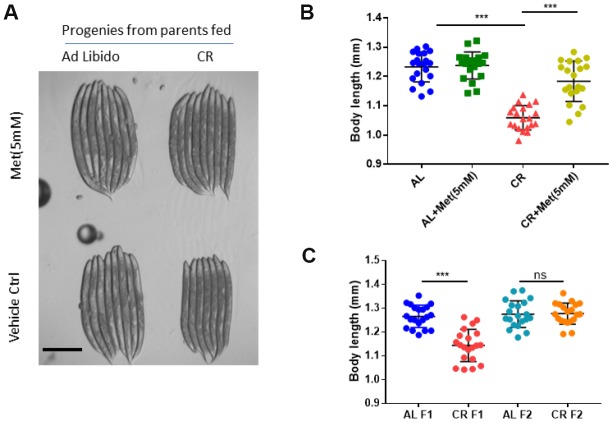
**Methionine supplementation reverses the negative effect of calorie restriction (CR) on small body size of the progenies.** (**A**) Representative image of progenies from worms raised under Ad libido (AL) and CR conditions. AL and CR were achieved by plating 1X10^11^/mL and 1X10^8^/mL OP-50 bacteria, respectively, on solid nematode growth (NG) medium containing carbenicillin and kanamycin. Parental worms were raised on NG medium supplemented with indicated concentration of methionine (Met) from hatching to day-1 adulthood. Worms were allowed to lay eggs for 2 hours to obtained synchronized embryos. Progenies were raised on NG medium with growing OP-50 to day-1 adulthood and imaged with Leica microscope. (**B**) CR reduces body size of progenies, which can be mitigated by methionine. Parental worms raised under Ad libido (AL) and CR conditions were treated with 5mM methionine (Met) as in A and progenies were collected and imaged as in A. Data were collected from 2 independent experiments. Body length of 20 worms were measured by using ImageJ and plotted by using Graphpad Prism software. P values were obtained by t-test: ***, P<0.0001. (**C**) The negative effect of CR on body size of progenies is transient. The first (F1) and second (F2) generations of progenies from parental worms raised under Ad libido (AL) and CR conditions were raised and imaged as in A and measured as in B. Data were collected from 2 independent experiments. P values were obtained by t-test: ns, not significant; ***, P<0.0001.

We also asked if progenies from the calorie-restricted animals could be defect in development and whether methionine can rescue such defect. To test this, we calorie restricted the parental animals from L1 stage as before and collected eggs within 3 hours to obtain age-synchronized progenies. Grown on normal NG medium with Ad libido OP-50 bacteria food, the progenies of calorie-restricted parents appeared to be smaller than controls (Figure 2A, 2B). Interestingly, adding methionine almost completely reversed the small body size. The slow growth phenotype only occurred transiently, as it disappeared in the second (F2) generation (Figure 2C).

### Methionine does not shorten the CR-induced long lifespan in *C. elegans*

**Figure 3:**
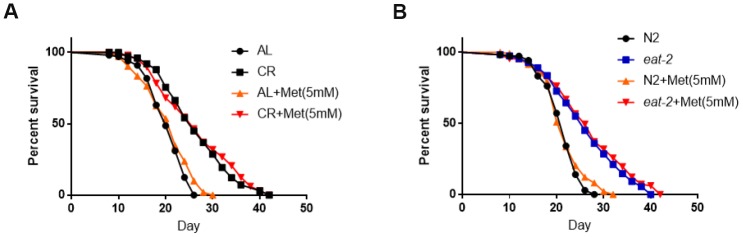
**Methionine does not shorten the long lifespan achieved by calorie restriction.** (**A**) The long lifespan from feeding diluted bacteria is not significantly affected by 5mM methionine. Ad libido (AL) and CR conditions were achieved by plating 1X10^11^/mL and 1X10^8^/mL OP-50 bacteria, respectively, on solid nematode growth (NG) medium containing carbenicillin and kanamycin. Worms were raised on NG medium supplemented with or without 5mM methionine (Met) throughout life with frequent transfer to new plates to avoid contamination by progenies. Number of deaths and lives were recorded every other day. Data from 3 independent experiment were pooled and plotted by using Graphpad Prism software. P values were obtained by log-rank test: CR (met) vs. CR, not significant; CR(met) vs. AL, P<0.0001. See Supplementary Table 1 in Supplemental Information (SI) for more information. (**B**) The long lifespan from CR model (*eat-2* mutant) is not significantly affected by 5mM methionine. N2 wild-type and *eat-2(ad1116)* worms were raised on normal NG medium with sufficient OP-50 bacterial food and also supplemented with and without 5mM methionine (Met). Number of deaths and lives were recorded every other day and with frequent transfer to new plates to avoid contamination by progenies. Data from 3 independent experiment were pooled and plotted by using Graphpad Prism software. P values were obtained by log-rank test: e*at-2*(met) vs. *eat-2*, not significant; *eat-2*(met) vs. N2, P<0.0001. See Supplementary Table 2 in Supplementary Information (SI) for more information.

Methionine has been implicated in lifespan regulation in many organisms and human cells [19]. It has therefore been proposed that methionine or its metabolite serve as a switch to control lifespan during CR. We asked if methionine supplemented in NG medium would cancel lifespan extension induced by CR. Fed diluted bacteria as the CR regimen, *C. elegans* exhibited extended lifespan as expected. Adding methionine at the optimal concentration that reversed reproduction defect did not obviously change the lifespan of either control animals or animals under CR condition (Figure 3A). Similarly, in *eat-2(ad1116)* mutant, the long lifespan was not obviously affected by 5mM methionine supplemented in NG medium (Figure 3B). Methionine had taken effect in our experiments as we noticed the same robust increase in the offspring body size as shown in Figure 2A. We concluded that methionine did not shorten the long lifespan of calorie-restricted animals. Our data also suggest that, at certain situations, CR extension of lifespan can be decoupled from its effect on reproduction.

**CR-induced reproduction defect in *C. elegans* is due to compromised oocyte but not sperm**

**Figure 4:**
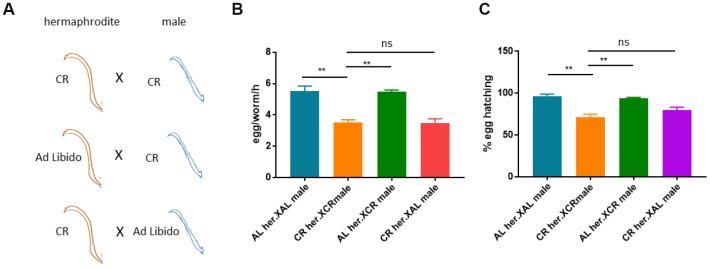
**Calorie restriction-induced reproduction defect is attributed to oocyte but not sperm.**(**A**) Schematic diagram showing the experimental design to study the origins of reproductive defect caused by CR. Shown are different crosses between CR hermaphrodites and males. CR hermaphrodites crossed to CR males is expected cto still have reproductive defect. If normal hermaphrodites crossed to CR males still have reproductive defect, then the defect was traced to sperm. If CR hermaphrodites crossed to normal males still have reproductive defect, then the defect was traced to oocyte. (**B**) Evaluation of egg production from different crosses shown in A. Males were maintained by crossing equal number of males to hermaphrodites. Ad libido (AL) and CR conditions were achieved by plating 1X10^11^/mL and 1X10^8^/mL OP-50 bacteria, respectively, on solid nematode growth (NG) medium containing carbenicillin and kanamycin. CR or non-CR males and hermaphrodites (n>20) were crossed for 1 day and hermaphrodites were transferred to new plate to collect synchronized eggs. Egg production were evaluated in per worm per hour (egg/worm/h). Data were collected from 3 independent experiments. P values were obtained by t-test: ns, not significant; **, P<0.001. (**C**) The hatching rate of different crosses shown in A. CR and crossed were conducted as in B and synchronized eggs were collected from 20 mated hermaphrodites and counted. Eggs were allowed to hatch for 2-3 days and successful hatching were determined by survival worms. Data were collected from 3 independent experiments with each experiment examine n>100 eggs. P values were obtained by t-test: ns, not significant; **, P<0.001.

*C. elegans* are majorly hermaphrodites and can reproduce with their own oocyte and sperm. Males are present at very low frequency and may increase under stress conditions, including calorie restriction [37]. We were interested to know if the defect in reproduction caused by CR was originated from oocyte, sperm or both. To this end, we calorie-restricted either hermaphrodites or males and cross them to *ad libido* (AL) males or hermaphrodite (Figure 4A). If CR- induced reproduction defect was due to sperm but not oocyte, we would expect to see (1) rescue of egg production from CR hermaphrodites crossed to AL males and (2) crossing CR males to AL hermaphrodites would reduce egg production. To the contrast, if due to oocyte but not sperm, (1) there would be no rescue of egg production by crossing CR hermaphrodites to AL males and (2) crossing CR males to AL hermaphrodites would not reduce egg production. If it was due to both oocyte and sperm, (1) either crossing CR hermaphrodites to normal males or crossing CR males to normal hermaphrodites would not rescue egg production and (2) crossing CR hermaphrodite to CR males should aggravate the defect in egg production. Our results showed that the defect in reproduction under CR condition was mostly originated from defect in oocyte but not sperm (Figure 4B and 4C).

**Methionine elevates the yolk proteins to mitigate CR’s negative effect on reproduction in *C. elegans***

**Figure 5:**
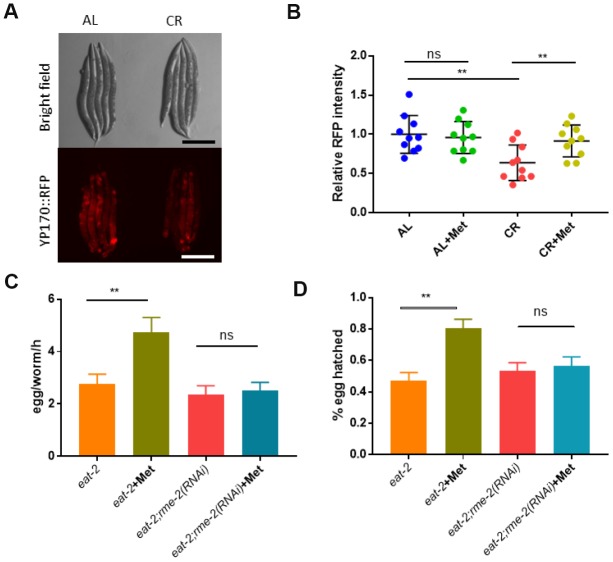
**The yolk proteins vitellogenin are required for methionine to mitigate the negative effect of CR on reproduction.** (**A**) Calorie restriction reduced yolk protein production. Animals expressing tdimer2 -tagged vitellogenin YP170 were raised under ad libido (AL) and calorie restricted (CR) conditions as shown in Figure 1A and imaged by using Leica microscope. Representative images of 2 independent experiments were shown. (**B**) Methionine reversed the decreased YP170::tdimer2 expression caused by CR Animals expressing tdimer2-tagged vitellogenin YP170 were raised under ad libido (AL) and calorie restricted (CR) conditions as shown in Figure 1A and imaged by using Leica microscope. 10 worms from 2 independent experiments were quantified by using ImageJ software and plotted by using Graphpad Prism software. P values were obtained by using student’s t-test: ns, not significant; **, P<0.001. (**C**) Blocking oocyte uptake of yolk proteins vitellogenin prevents methionine’s beneficial effects on CR-compromised egg production. CR were achieved by using the *eat-2(ad1116)* mutant worms. N2 wild-type and *eat-2* mutant worms were raised on HT115 bacteria expressing vector only control (L4440) or *rme-2* double stranded interference RNA (RNAi) to knock down *rme-2* gene expression. Day-1 adult worms were examined for egg production by counting the total eggs within 3 hours. Data were collected from 3 independent experiment and plotted to show egg from per worm per hour (egg/worm/h). P values were obtained by t-test: ns, not significant; **, P<0.001. (**D**) Blocking oocyte uptake of yolk proteins vitellogenin prevents methionine’s beneficial effects on CR-compromised embryo survival. CR and RNAi of N-2 wild-type and *eat-2* animals were conducted as in E and synchronized eggs (n>100) were obtained by allowing day-1 adult worms to lay eggs for 2 hours. Survival were measured by counting the dead and viable embryos. Data from 3 independent experiments were collect for analysis. P values were obtained by t-test: ns, not significant; **, P<0.001.

We explored the molecular pathways that mediate CR’s negative effect on reproductive ability. Since CR only affected oocyte fertility, we focused on gene expression and metabolites specific to oocyte. One of the most obvious differences between oocyte and sperm is the yolk protein, which is essential for oocyte maturation and embryonic development but is not known to be expressed in sperm of *C. elegans*. We tested if yolk protein production was affected by CR (*eat-2* mutant) and whether it could be rescued by methionine. Vitellogenin are major yolk proteins, which are expressed from 6 *vit* genes in *C. elegans* [38]. By using a transgenic strain expressing a tdimer2-tagged vitellogenin (YP170::tdimer2) as an indicator for yolk proteins [39], we found that CR animals showed significant reduction in yolk protein expression, which was rescued by supplementing methionine in the food (Figure 5A and 5B). Methionine did not increase the YP170::tdimer2 in wild-type animals, suggesting a specific effect of methionine on yolk proteins (Figure 5B).

Next, we asked if yolk proteins were required for the methionine to rescue the reproductive defect of CR animals. Yolk proteins are made in the intestine and transported into the oocyte to support embryonic growth and survival. Yolk proteins uptake by oocytes requires endocytosis mediated through RME-2 [40]. We found that RNAi knockdown of *rme-2* expression in the *eat-2* CR model animals prevented methionine from improving egg production (Figure 5C) and hatching rate (Figure 5D). These results suggest that increased yolk production and transport into the oocyte is required for methionine to improve the impaired reproduction of CR animals.

## DISCUSSION

Our study in *C. elegans* shows that CR/DR could cause detrimental effect on the reproductive system. CR/DR significantly reduces the number of eggs and viable embryos. Importantly, CR/DR also negatively affects the development and growth of the offspring. The negative effect of CR/DR on reproduction and its transgenerational property suggest important message to those practicing CR/DR for health benefits. We trace the origin of the side effect to be eggs rather than sperm The dimorphism of sex response to CR is consistent with reports showing that CR does not extend lifespan of males [41] and glucose-rich medium shortens. lifespan of hermaphrodites but not males [42] Interestingly, methionine supplementation can reverse all the defects observed in the reproductive systems in *C. elegans*.

Despite its rescuing effect, methionine does not cancel the extended lifespan achieved by CR in C. elegans, suggesting that the benefits of CR on lifespan extension can be decoupled from its negative effects on reproduction and growth. These results are consistent with previous studies in Drosophila [31, 43], where fecundity and survival under certain CR conditions can be unlinked, suggesting that methionine could be a potential food supplement for humans practicing CR. It will be also interesting to know if in higher animals methionine could have beneficial effect on CR-related diseases such as immunity defect [44, 45] and osteoporosis [46].

Interestingly, we found that the side effect of CR could be transgenerational. Offspring of calorie-restricted animals not only are smaller in size than controls, but also develop slower. These results are consistent with some early studies in human showing the negative effect of long-term undernutrition birth weight, head circumference and mental and physical health in early adult [16–18]. The transgenerational effect of calorie restriction has also been reported for lifespan extension, where evidence points to epigenetic alternations in early embryos of calorie-restricted parents [47, 48]. The transgenerational effects become indiscernible in later generations in our study (Figure 2) and previous reports [47, 48], supporting the involvement of epigenetic alternation rather than genetic selection. Considering the importance of maternal nutrition on embryos, further investigations into the transgenerational side effect of CR, especially on higher animals, are urgently needed.

How does methionine rescue CR-induced reproductive defect? In addition to functioning as the first amino acid in polypeptides, methionine also regulates DNA methylation and antioxidant balance [49]. Modulation of genes in the methionine metabolisms can protect embryonic stem cells from oxidative stress [50], however, a direct role of antioxidant effect on embryonic development has not been reported. Interestingly, we find that CR can reduce the expression of egg yolk protein YP170 in the intestine, which is rescued by methionine supplementation (Figure 5). Importantly, such rescue is blocked by a mutation [40] that blocking egg yolk protein transport from intestine to the oocyte (Figure 5), suggesting that methionine’s beneficial effect on reproduction could be at least partly attributed to enhanced egg yolk production. As yolk protein is essential for oocyte maturation, CR might selectively reduce egg yolk proteins in the intestine, which in turn cause all the phenotypes of reproductive impairment, including egg laying, hatching rate and offspring growth. On the opposite, ad libido C. elegans produces and accumulates yolk proteins by converting its intestinal biomass to yolk proteins, favoring reproduction but interestingly causing multiple aging phenotypes [51]. Together, our study supports the “hyperfunction theory of aging” where continued growth and increased reproduction can lead to aging and age-related pathologies [52].

How does methionine modulate gene expression such as those involved in yolk protein production? Maternal nutrition can modulate epigenetics in the fetal genome through DNA methylation, resulting in permanent structural and physiological alternations in the offspring [1, 2, 53]. DNA methylation requires S-adenosylmethionine (SAM) as the major methyl group donor in the cell, which is derived largely from Met, in addition to betaine, choline, and 5-methyltetrahydrofolate (5-MTHF) [54, 55]. Since methionine is an essential methyl group donor for DNA methylation, the rescuing effect of methionine could be due to epigenetic changes favoring gene expression for embryonic maintenance. Indeed, transcriptional profiling of embryos from bovine preimplantation embryos shows that methionine suppress general gene expression [56], consistent with its role in DNA methylation. Methylation of DNA and selective gene transcription is likely very important for embryonic maintenance, as methionine metabolisms are elevated in embryonic stem cells from mouse and human and lacking methionine renders embryonic cells sensitive to apoptotic cell death [54]. Whether methionine’s beneficial effect on reproduction under CR involves epigenetic regulation, however, await further investigations.

## MATERIALS AND METHODS

Please refer to Supplementary Information for additional and detailed protocols.

### Strains and medium

Transgenic vitellogenin reporter strain RT368 (pwIs98 [YP170::tdimer2 + unc-119(+)]) were published before [39], which were crossed to the control strain (N2 Bristol wild-type) 3 times. Standard nematode growth medium (NGM) were prepared according to Wormbook (http://www.wormbook.org/chapters/www_strainmaintain/strainmaintain.html). Carbenicillin (50 μM) and Kanamycin (50 μM) were added to NG medium before pouring agar plates. Methionine were dissolved in water and added to NG medium before pouring agar plates. *C. elegans* strains were maintained at 20 °C on standard NGM plates seeded with OP-50 bacteria. For calorie restriction, OP-50 bacteria were cultured and diluted into 1X10^11^/ml and 1X10^8^/ml then plated on agar plate as shown in [35].

### RNAi treatment

RNAi experiments was conducted by feeding worms on agar plates with bacteria expressing double-stranded RNA (dsRNA) for *rme-2*. RNAi clones were originally from [57]. Specifically, RNAi bacteria were cultured to log phase and seeded on NG plates containing 50 ug/mL Carbenicillin and 1 mM Isopropyl β-D-1-thiogalactopyranoside (IPTG) for at least 24 hours to induce dsRNA expression. L1 stage worms were then transferred to and maintained on the RNAi plate for gene knockdown experiments.

### Egg production and survival assay

Worms were cultured on ad libido (AL) or calorie restricted (CR) medium from hatching to day-1 adulthood. AL and CR conditions were achieved by plating 1X10^11^/mL and 1X10^8^/mL OP-50 bacteria, respectively, on solid nematode growth (NG) medium containing carbenicillin and kanamycin. Worms were transferred to normal NG medium plates (5 worms/plate) for 3 hours to allow egg laying. Eggs were counted and divided by time and number of parental worms to obtain egg/worm/h. For embryo survival experiment, eggs collected above where allowed to hatch to L1/L2 worms. The numbers of larvae and dead eggs were counted. Experiments were repeated in 3 different days and data were collected for analysis by GraphPad Prism software.

### Quantification of worm length and fluorescence

Synchronized eggs from AL and CR parental hermaphrodites were collected and allowed to hatch and develop to day-1 adulthood. Worms were picked randomly and imaged with Leica stereomicroscope equipped with fluorescence channel. To quantify the length of worms, ImageJ software were used to draw lines in the middle of the worms from head to tail and measure to distance. For fluorescence intensity, ImageJ software were used to measure the area and the fluorescent signals of individual worms. The fluorescent intensity of individual worms was obtained by dividing the signals by worm area. Data were obtained from 10-20 animals and relative intensity were plotted with GraphPad Prism software by normalizing to the average value of controls.

### Lifespan assay

For lifespan assay in *C. elegans*, gravid worms were allowed to lay synchronized eggs on AL and CR plate supplemented with or without 5mM methionine for 2 hours (5 worms per plate). Eggs were allowed to hatch at 20°C young adulthood and worms were transferred to new plates every day to keep contamination by progenies. Lifespan were started from day-8 of adulthood by counting the survival and dead worms every other day. Worms with explosion, bagging and protruding vulva were censored. Death was defined by lack of any visible movement for 5 seconds after touching the tail. Lifespan data were also shown in Supplemental Information (SI). Lifespan assays were performed at different time for 3 times and pooled together to be plotted and analyzed with GraphPad Prism software.

### Mating experiment

Mating was conducted by raising 5 males and 5 hermaphrodites at young adult stages on NG agar medium plate with a tiny spot of OP-50 bacteria (mating plate). To obtain age-matched CR males and hermaphrodites, hermaphrodites mated for 1 day were transferred to CR and AL plates to lay synchronized eggs for 2 hours. Eggs were allowed to hatch and develop to L4 larvae. Age-matched males and hermaphrodite were picked for mating on mating plate according to the combinations shown in Figure 4A.

## Supplementary Material

Supplementary Methods

Supplementary Figures

Supplementary Tables
