# Supplementary material for: Methionine increases yolk production to offset the negative effect of caloric restriction on reproduction without affecting longevity in C. elegans
Source: Aging (Albany NY). 2020 Feb 6;12(3):2680–97. doi: 10.18632/aging.102770 (PMC7041781; doi:10.18632/aging.102770)
Supplement: Supplementary Methods [file aging-12-102770-s002..pdf]

SUPPLEMENTARY METHODS

NG Medium

(1) 1M Potassium Phosphate buffer, pH 6.0: Dissolve in 1L distilled water 108.3 g KH_2PO_4, 35.6 g K_2HPO_4-H_2O, autoclave at 121 °C for 20 min.

(2) Make 1 M $MgSO_4$, 1 M $CaCl_2$ stock solution by dissolving in distilled water and autoclaving at 121 °C for 20 min.

(3) Prepare 5 mg/mL Cholesterol in 98% ethanol, filtered through 2.2 µm solvent compatible PVDF syringe filter.

(4) Prepare 100mg/mL carbenicillin and 50mg/mL kanamycin stock solution in water, filtered through 2.2 µm syringe filter.

(5) Nematode Growth Media (NGM), 1L: dissolve 3 g NaCl, 2.5 g Bacto-Peptone and 20g Agar to distilled water, autoclave at 121 °C for 20 min. When cool down to around 60 °C, add 1mL $MgSO_4$(1 M), 1mL $CaCl_2$(1 M), 1mL Cholesterol (5 mg/mL), 25mL Potassium Phosphate buffer (1M). Mix thoroughly. Pipette 4 mL to 60mm culture dish.

(6) To make RNAi plate, 1mL carbenicillin (100mg/mL) and 1 mM Isopropyl β-D-1-thiogalactopyranoside (IPTG) were also added before pouring the medium to the plate in step (5).

(7) To make CR/DR plate, 1mL carbenicillin (100mg/mL) and 1mL kanamycin (50mg/mL) were added before pouring the medium to the plate in step (5).

Amino acid supplementation

(1) According to sigma product specification, the solubility of L-Cysteine hydrochloride monohydrate (C7880) in H_2O is up to 50 mg/ml (284.69mM). To make 200mM stock solution, dissolve 1.76 g L-Cysteine hydrochloride monohydrate in ddH_2O and autoclave at 121 °C for 20 min.

(2) According to sigma product specification, the solubility of n-acetyl-Cysteine in water is up to 100mg/ml (612.78mM). To make NAC stock solution of 200mM, 1.63 g NAC powder (Sigma Catalog No. A7250) was dissolved in 50 mL ddH_2O with stirring and heating until clear then autoclaved at 121 °C for 20 min

(3) According to sigma product specification, the solubility of L-Threonine in water is up to 50 mg/ml (419.74mM). To make NAC stock solution of 200mM, 1.19 g L-Threonine powder (Sigma Catalog No. T8625) was dissolved in 50 mL ddH_2O with stirring and heating until clear then autoclaved at 121 °C for 20 min

(4) As L-Leucine has limited solubility in water, we use 100mM L-Leucine hydrochloride solution from Sigma (Sigma Catalog No.80687).

(5) NG medium were prepared, autoclaved and cooled down to around 60 °C, then amino acid solutions were added to make final concentrations of 1mM, 2mM, 5mM, 10mM. The stock solution was used within 3 month and the plates were used within 1 month.

Seeding bacteria

(1) OP-50 bacteria were maintained on LB broth. Single colony was picked and cultured in 10 mL LB broth by shaking at 37°C incubator overnight.

(2) To obtain a standard curve for the relationship between OD600 and the concentration of bacteria (cfu/ml), bacteria were diluted 2x, 4x, 8x, 16x, 32x and the OD600 were determined. Bacteria at each dilution were further diluted 1X106 times (1000-fold dilution twice) and 0.1 ml were plated on a LB agar plate. After overnight incubation at 37°C, count the number of bacterial colonies and convert the concentration to (number of bacteria/ml), which is the same as cfu/ml. The relationship between measured OD600 values and OP50 bacteria concentrations can be established by plotting OD600 as a function of bacterial concentration (cfu/mL). Linear regression was obtained by using Excel software.

(3) To prepare control and CR/DR medium plate, the OD600 values of fresh OP-50 culture corresponding to $1x10^{11}$/ml (control) and $1X10^{8}$/ml (CR/DR) were determined from standard curve in previous step. 0.2ml of bacteria were added to the center of 60mm NGM plate containing 100mg/mL carbenicillin and 50mg/mL kanamycin to prevent bacteria from growing. Place plates in 37 °C incubator to dry the bacteria

culture. Plate can be stored at 4°C for at least 1 months.

RNAi treatment

(1) RNAi agar plates were prepared as show in in above, "NG medium, step (6)".
(2) RNAi bacteria. RNAi clones and vector only control were picked from a bacterial library and cultured in LB liquid medium supplemented with 50mg/ml carbenicillin at 37 °C with vigorous shaking overnight. Dilute RNAi bacteria culture to OD600 ~ 0.2 and continue to culture at the same condition for ~ 2 hours until the OD600 ~ 0.5.

(3) Add RNAi bacteria to RNAi agar plate shown in (1) in the middle of the plate, avoid touching the surface of agar medium. Let it dry at room temperature (~25°C) for at least 24 hours to induce dsRNA expression. Plates can be stored at 4 °C for at least 1 month. Always use the same batch for control and treated groups.

(4) Preparing synchronized worms. Worms were maintained at 20 °C on NG agar medium by transferring about 10 asynchronized larvae worms to fresh plate every 3~4 days. To obtain age synchronized L1 animals, gravid worms were transferred to RNAi bacteria plate (5 worms/plate) for 3 hours to allow egg laying. Then gravid worms were removed from the plates.

(5) Transfer worms to new plate. Eggs on RNAi plates were maintained at 20 °C for about 3 days where animals reach adult stage. Transfer animals to new plate every 2 days until animals a longer lay eggs.

Egg production and hatching assay

(1) CR/DR treatment. CR/DR plates were prepared as show in "NG medium, step (6)". Worms were normally maintained at 20 °C on NG agar medium by transferring about 10 asynchronized larvae worms to fresh plate every 3~4 days to avoid starvation. To CR/DR the animals, gravid worms were transferred to control (ad libido or AL) or CR/DR plate (5 worms/plate) for 3 hours to allow egg laying. Then gravid worms were removed from the plates.

(2) Synchronizing eggs. Worms were cultured on ad libido (AL) or calorie restricted (CR) medium from hatching to day-1 adulthood. Gravid worms from both AL and CR conditions were transferred to ad libido (AL) NG medium plates (5 worms/plate) for 3 hours to allow egg laying. Since parental worms at CR conditions grow slower, to do the egg production assay at the same time, do step (1) for several days in a row to make sure the gravid worms are available for both CR and AL condition. Eggs were counted after 3 hours and divided by time and number of parental worms to obtain egg/worm/h. Experiments were repeated 3 times and data were collected for analysis by GraphPad Prism software.
(3) For embryo survival experiment, eggs collected above where allowed to hatch overnight. The numbers of larvae and dead eggs were counted. Experiments were repeated in 3 different days and data were collected for analysis by GraphPad Prism software.

Quantification of worm length and fluorescence by ImageJ

(1) Synchronized eggs from AL and CR. Parental worms were cultured on ad libido (AL) medium plate until adulthood. Gravid worms were transferred to control (ad libido or AL) or CR/DR plate (5 worms/plate) for 3 hours to obtain synchronized eggs. Then gravid worms were removed from the plates. Since worms at CR conditions grow slower, to obtain age-similar young adult worms, do egg synchronization for several days in a row to make sure the young adults are available for both CR and AL condition.

(2) Synchronized eggs from step (1) were allowed to grow at 20 °C until adulthood. Day-1 adult animals were picked randomly and imaged with Leica stereomicroscope equipped with fluorescence channel. Experiments were repeated biologically at least for 3 times at different dates.

(3) ImageJ software were used to quantify the length of the animals. Draw lines in the middle of the worms from head to tail and measure to distance, which is indication of the length of the animals. Do the same for ~ 20 animals from different image panel for each condition and plot the relative length of worms with GraphPad Prism software by normalizing to the average length of control animals.

(4) For fluorescence intensity, synchronized eggs were obtained and animals raised as in step (1) and (2). ImageJ software were used to measure the area and the fluorescent signals of individual worms. The fluorescent intensity of individual worms was obtained by dividing the signals by worm area. Data were obtained from 10-20 animals and relative intensity were plotted with GraphPad Prism software by normalizing to the average value of controls

Lifespan assay of adult animals

(1) NG medium plates of CR and AL, with and without Methionine were prepared as shown in “NG medium, step (6) and (7)”.

(2) To prepare age-synchronized worms, gravid worms from AL plate were allowed to lay eggs on AL and CR plate supplemented with or without 5mM methionine for 2 hours (5 worms per plate).

(3) To maintain adult worms, eggs were incubated at 20 °C incubator until adulthood. Adult worms were transferred to new plate to avoid overcrowded by larvae. Keep transfer animals every other day until no eggs were produced.

(4) For lifespan assay in *C. elegans*, lifespan was started from day-8 of adulthood by counting the survival and dead worms every other day. Worms with explosion, bagging and protruding vulva were censored. Death was defined by lack of any visible movement for 5 seconds after touching the tail. Lifespan data were also shown in Supplementary Information (SI) Supplementary Tables 1 and 2. Lifespan assays were performed at different time for 3 times and pooled together to be plotted and analyzed with GraphPad Prism software.

Mating assay

(1) Obtaining males. *C. elegans* males presents in the AL condition less than 0.5%. To obtain males, L4 hermaphrodite were picked to AL NG plate (4 worms/plate, 5 plates) and incubated at 30 °C for 5 hours. Heat-shocked animals were transferred back to 20 °C incubator for ~3 days. Males were identified and picked to new NGM plates.

(2) Keeping male population. Maintain male populations by picking 5 males and 5 hermaphrodites to mating plates. The mating plates are generated by transferring a tiny bit of OP-50 bacteria on the middle of empty NGM medium plates.

(3) Obtaining age-matched males and hermaphrodites from AL and CR condition. Pick gravid hermaphrodites from mating plates in (2) to AL and CR plates (5 worms/plate, 5 plates). Allow egg laying for 2 hours. Remove hermaphrodites from plates. Incubate at 20 °C for ~3 days. Identify and separate L4 males and hermaphrodite from AL and CR conditions to new plates.

(4) Mating of CR and AL animals. Pick L4 males and hermaphrodites from AL and CR conditions on mating plate as shown in (2), according to the combinations shown in (2) Figure 4A. Animals were allowed to mate overnight.

(5) Egg production and hatching assay of mated animals. Mated hermaphrodites were transferred to normal NGM plates for 2 hours to collect synchronized eggs. Egg production and hatching rate were conducted as shown in “Egg production and hatching assay step (2)-(3)”.
