# Supplementary material for: Methionine increases yolk production to offset the negative effect of caloric restriction on reproduction without affecting longevity in C. elegans
Source: Aging (Albany NY). 2020 Feb 6;12(3):2680–97. doi: 10.18632/aging.102770 (PMC7041781; doi:10.18632/aging.102770)
Supplement: Supplementary Figures [file aging-12-102770-s003..pdf]

SUPPLEMENTARY FIGURES

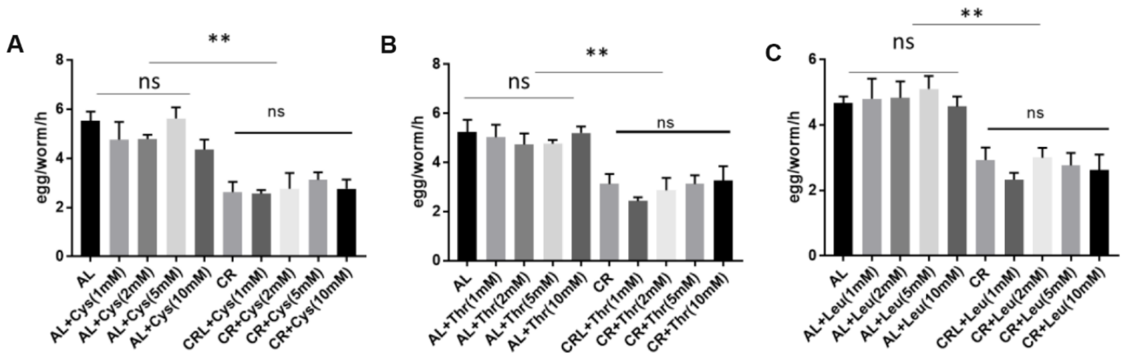

Supplementary Figure 1. Amino acids cysteine (**A**), threonine (**B**) and Leucine (**C**) did not improve the impaired egg production caused by calorie restriction (CR). Ad libido (AL) and CR conditions were achieved by plating 1X1011/mL and 1X108/mL OP-50 bacteria, respectively, on solid nematode growth (NG) medium containing carbenicillin and kanamycin. Worms were raised on NG medium supplemented with indicated concentrations of cysteine (Cys), threonine (Thr) and leucine (Leu) from hatching to day-1 adulthood. Worms (n>25) were allowed to lay eggs for 3 hours and egg production were evaluated in per worm per hour (egg/worm/h). Data were collected from 3 independent experiments. P values were obtained by t-test: ns, not significant; **, P<0.001.

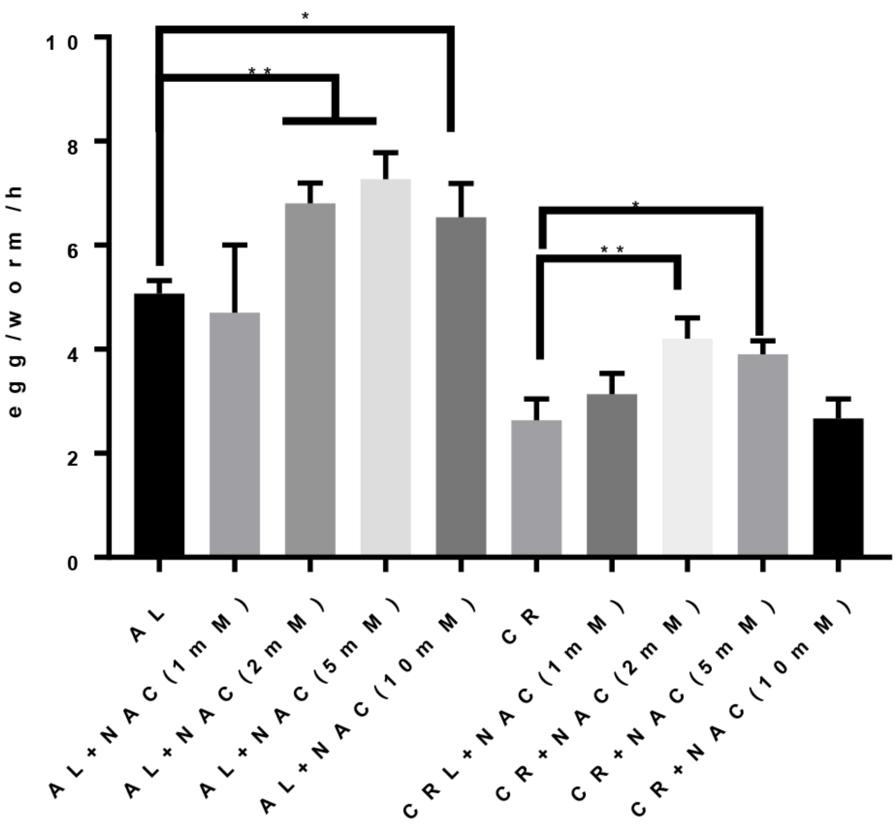

Supplementary Figure 2. The cysteine precursor n-acetyl-cysteine (NAC) enhance egg production in both control and calorie-restricted *C. elegans*. Ad libido (AL) and CR conditions were achieved by plating 1X1011/mL and 1X108/mL OP-50 bacteria, respectively, on solid nematode growth (NG) medium containing carbenicillin and kanamycin. Worms were raised on NG medium supplemented with indicated concentrations of NAC from hatching to day-1 adulthood. Worms (n>25) were allowed to lay eggs for 3 hours and egg production were evaluated in per worm per hour (egg/worm/h). Data were collected from 3 independent experiments. P values were obtained by t-test: *, P<0.01; **, P<0.001; otherwise, not significant.
