# Supplementary material for: Methionine increases yolk production to offset the negative effect of caloric restriction on reproduction without affecting longevity in C. elegans
Source: Aging (Albany NY). 2020 Feb 6;12(3):2680–97. doi: 10.18632/aging.102770 (PMC7041781; doi:10.18632/aging.102770)
Supplement: Supplementary Tables [file aging-12-102770-s001..pdf]

SUPPLEMENTARY TABLES

Supplementary Table 1. Related to Figure 3A.

**Treatment**	**Death**	**Censored**	**Median survival (day)**	**P value to Ctrl**	**P values to CR**
Ctrl	97	6	20	NA	<0.0001
Ctrl+Met(5mM)	101	6	22	NS	<0.0001
CR	98	9	26	<0.0001	NA
CR+Met(5mM)	97	9	26	<0.0001	NS

Supplementary Table 2. Related to Figure 3A.

**Treatment**	**Death**	**Censored**	**Median survival (day)**	**P value to N2**	**P values to *eat-2***
N2	100	13	22	NA	<0.0001
N2+Met(5mM)	99	14	22	NS	<0.001
eat-2	109	7	26	<0.0001	NA
eat-2+Met(5mM)	98	14	26	<0.0001	NS
